# Supplementary material for: Observation of perfect absorption in hyperfine levels of molecular spins with hermitian subspaces
Source: Nat Commun. 2025 Dec 10;17:470. doi: 10.1038/s41467-025-67163-z (PMC12800288; doi:10.1038/s41467-025-67163-z)
Supplement: Supplementary file 1 — Supplementary Information [file 41467_2025_67163_MOESM1_ESM.pdf]

SUPPLEMENTARY INFORMATION OF:
Observation of Perfect Absorption in Hyperfine Levels of Molecular Spins with Hermitian Subspaces

Claudio Bonizzoni[1,2*], Daniele Lamberto[3], Samuel Napoli[3], Simon Günzler[4], Dennis Rieger[4], Fabio Santanni[5], Alberto Ghirri[2], Wolfgang Wernsdorfer[4], Salvatore Savasta[3], Marco Affronte[1,2]

[1*]Department of Physics, Informatics and Mathematics, University of Modena and Reggio Emilia, via G. Campi 213/a, Modena, 41125, Italy.
[2]Institute of Nanoscience (NANO), National Research Council (CNR), via G. Campi 213/a, Modena, 41125, Italy.
[3]Department of Mathematics and Computer Sciences, Physical Sciences and Earth Sciences, University of Messina, Via Salita Sperone, c.da Papardo, Messina, 98166, Italy.
[4]Institute of Physics, Karlsruhe Institute of Technology, Wolfgang-Gaede-Str. 1, Karlsruhe, D-76131, Germany.
[5]Department of Chemistry Ugo Schiff, University of Florence, via della Lastruccia 3, Sesto Fiorentino (FI), 50019, Italy.

*Corresponding author(s). E-mail(s): claudio.bonizzoni@unimore.it;

Contents

1 Quantum Langevin Equations for the multiple spin ensemble coupled to the electromagnetic resonator 2

2 Additional Experimental Details 3
2.1 Resonators and Cryostat . . . 3
2.2 Estimation of the microwave photon number . . . 5

3 Additional Data 6
3.1 Data for empty resonators . . . 6
3.2 Tuning antenna position on BDPA . . . 8
3.3 Additional Data for VOTPP . . . 9

4 Fit Parameters for all data sets 11
4.1 Parameters for BDPA . . . 11
4.2 Parameters for VOTPP . . . 12

1 Quantum Langevin Equations for the multiple spin ensemble coupled to the electromagnetic resonator

Here, we derive the quantum Langevin equations leading to the reflection scattering parameter in Eq. (3) of the main text. The total Hamiltonian $\hat{H}_T$ for either the VOTPP or the BDPA sample, including their interaction with the resonator and all the reservoir channels, is given by:

$$\hat{H}_T = \hat{H}_S + \hat{H}_B + \hat{H}_I. \tag{S.1}$$

The system Hamiltonian $\hat{H}_S$ is provided in Eq. (1) of the main text. The bare reservoir Hamiltonian is given by

$$\hat{H}_B = \hbar \int_{-\infty}^{+\infty} d\omega\, \omega \left(\sum_{\mathrm{k=r,nr}} \hat{c}_{\mathrm{k}}^{\dagger}(\omega)\hat{c}_{\mathrm{k}}(\omega) + \sum_{\mu=1}^{N} \hat{d}_{\mu}^{\dagger}(\omega)\hat{d}_{\mu}(\omega) \right), \tag{S.2}$$

where $\hat{c}_{\mathrm{nr}}$ and $\hat{c}_{\mathrm{r}}$ are the bosonic annihilation operators associated with the internal and radiative losses of the resonator, respectively. Note that $\hat{d}_\mu$ represents the annihilation operator describing the reservoir field of the μ-th spin ensemble. Finally, the interaction of the system and the resonator with their corresponding reservoirs is described by (under the rotating wave approximation, RWA) [1, 2]:

$$\hat{H}_I = i\hbar \int_{-\infty}^{+\infty} d\omega \left[\sum_{\mathrm{k=r,nr}} \sqrt{\frac{\gamma_{\mathrm{k}}}{2\pi}} \left(\hat{c}_{\mathrm{k}}(\omega)\hat{a}^{\dagger} - \hat{c}_{\mathrm{k}}^{\dagger}(\omega)\,\hat{a}\right) + \sum_{\mu=1}^{N} \sqrt{\frac{\gamma_{\mu\mathrm{s}}}{2\pi}} \left(\hat{d}_{\mu}(\omega)\hat{b}_{\mu}^{\dagger} - d_{\mu}^{\dagger}(\omega)\hat{b}_{\mu}\right) \right], \tag{S.3}$$

where the decay rates are defined in the main text. Following a standard approach to open quantum systems [1, 2], we derive a set of quantum Langevin equations by substituting the solutions of the reservoir operators into the Heisenberg equations of motion for the system operators (the spin ensemble and the resonator):

$$\frac{d}{dt}\hat{\boldsymbol{\alpha}}(t) = -i\mathbf{A}\hat{\boldsymbol{\alpha}}(t) - \frac{1}{2}\boldsymbol{\Gamma}\hat{\boldsymbol{\alpha}}(t) + \hat{\mathbf{F}}_{\mathrm{in}}(t)\,, \tag{S.4}$$

$$\frac{d}{dt}\hat{\boldsymbol{\alpha}}(t) = -i\mathbf{A}\hat{\boldsymbol{\alpha}}(t) + \frac{1}{2}\boldsymbol{\Gamma}\hat{\boldsymbol{\alpha}}(t) + \hat{\mathbf{F}}_{\mathrm{out}}(t)\,. \tag{S.5}$$

In the last expressions, we introduced the block matrices

$$\hat{\boldsymbol{\alpha}} = \begin{pmatrix} \hat{a}(t) \\ \hat{b}_1(t) \\ \vdots \\ \hat{b}_N(t) \end{pmatrix}, \; \mathbf{A} = \begin{pmatrix} \omega_0 & \mathbf{g}^T \\ \mathbf{g} & \boldsymbol{\omega_s} \end{pmatrix}, \; \boldsymbol{\Gamma} = \begin{pmatrix} \gamma_{\mathrm{r}} + \gamma_{\mathrm{nr}} & 0 \\ 0 & \boldsymbol{\gamma_s} \end{pmatrix}, \tag{S.6}$$

where $\boldsymbol{\omega_s}$ and $\boldsymbol{\gamma_s}$ are diagonal block matrices with the μ-th elements given by $\omega_{s\mu}$ and $\gamma_{s\mu}$ (where μ ranges from 1 to N), respectively. Additionally, we defined

$$\boldsymbol{g}^T = \big(g_1, ..., g_N\big) \ , \tag{S.7}$$

and the input and output field vectors as

$$\hat{\mathbf{F}}_{\text{in}}(t) = \begin{pmatrix} \sqrt{\gamma_{\text{r}}}\hat{a}_{\text{r,in}}(t) + \sqrt{\gamma_{\text{nr}}}\hat{a}_{\text{nr,in}}(t) \\ \sqrt{\gamma_{\text{s}\mu}}\,\hat{b}_{1,\text{in}}(t) \\ \vdots \\ \sqrt{\gamma_{\text{s}N}}\,\hat{b}_{N,\text{in}}(t) \end{pmatrix} , \ \hat{\mathbf{F}}_{\text{out}}(t) = \begin{pmatrix} \sqrt{\gamma_{\text{r}}}\hat{a}_{\text{r,out}}(t) + \sqrt{\gamma_{\text{nr}}}\hat{a}_{\text{nr,out}}(t) \\ \sqrt{\gamma_{\text{s}\mu}}\,\hat{b}_{1,\text{out}}(t) \\ \vdots \\ \sqrt{\gamma_{\text{s}N}}\,\hat{b}_{N,\text{out}}(t) \end{pmatrix} . \tag{S.8}$$

The components of the input vector are given by

$$\begin{aligned} \hat{a}_{\text{r,in}}(t) &= \frac{1}{\sqrt{2\pi}} \int_{-\infty}^{+\infty} d\omega\, e^{-i\omega\,(t-t_0)} \hat{c}_{\text{r}}(\omega; t_0) \ , \\ \hat{a}_{\text{nr,in}}(t) &= \frac{1}{\sqrt{2\pi}} \int_{-\infty}^{+\infty} d\omega\, e^{-i\omega\,(t-t_0)} \hat{c}_{\text{nr}}(\omega; t_0) \ , \\ \hat{b}_{\mu,\text{in}}(t) &= \frac{1}{\sqrt{2\pi}} \int_{-\infty}^{+\infty} d\omega\, e^{-i\omega\,(t-t_0)} \hat{d}_{\mu}(\omega; t_0) \ . \end{aligned} \tag{S.9}$$

where $t_0 < t$ is the initial time (input) chosen to solve the equations of motion for the reservoir field operators. Similarly, the output field vector entries have the same form as in Eq. (S.9), except that t_0 is replaced by t_f ($t < t_f$, output). Rewriting the Langevin equations Eq. (S.4) and Eq. (S.5) in the frequency domain allows us to express $\mathbf{F}_{\text{out}}(\omega)$ in terms of $\mathbf{F}_{\text{in}}(\omega)$:

$$\hat{\mathbf{F}}_{\text{out}}(\omega) = \big[-\frac{1}{2}\mathbf{\Gamma} - i(\omega\,\mathbf{I} - \mathbf{A})\big]\,\big[\frac{1}{2}\mathbf{\Gamma} - i(\omega\,\mathbf{I} - \mathbf{A})\big]^{-1}\,\hat{\mathbf{F}}_{\text{in}}(\omega)\,, \tag{S.10}$$

where $\mathbf{I}$ is the identity matrix. Moreover, the input-output relationships are derived by subtracting Eq. (S.4) to Eq. (S.5) in the frequency domain, namely

$$\hat{\mathbf{F}}_{\text{out}}(\omega) = -\mathbf{\Gamma}\hat{\boldsymbol{\alpha}}(\omega) + \hat{\mathbf{F}}_{\text{in}}(\omega)\,. \tag{S.11}$$

From this equation follows the input-output relation for the radiative port, $\hat{a}_{\text{r,out}}(\omega) = \hat{a}_{\text{r,in}}(\omega) - \sqrt{\gamma_{\text{r}}}\,\hat{a}(\omega)$, which is used to calculate the complex scattering coefficient (see Methods).

2 Additional Experimental Details

2.1 Resonators and Cryostat

Each chip used in the experiments is made of sapphire with dimensions $3 \times 10\,\text{mm}^2$ and thickness 420 μm. The short side of the chip carries two planar microwave

resonators, namely Res #1 and Res #2, working in reflection mode and coupled to the same feeding antenna, whose position can be adjusted at room temperature, as in Suppl. Fig. 1 and Fig. 1 of the main text. The two resonators are made of superconducting Niobium films (thickness, 50 nm). The geometry is a lumped-element LC resonator, in which the interdigitated part acts as a large capacitance, and the central loops give a small inductance, giving large microwave currents around the loop position. The width of the superconducting niobium strip is $w = 10\,\mu$m, while the interspace between each part of the capacitors is $w' = 20\,\mu$m. The length of the resonator is $l_1 = 1$ mm, while its distance from the edge of the chip is $d_1 = 190\,\mu$m. Resonators #1 and #2 are designed to have two fundamental resonant frequencies $\omega_{0,1}/2\pi = \nu_{0,1} \approx 9.9\,$GHz and $\omega_{0,2}/2\pi = \nu_{0,2} \approx 11\,$GHz, respectively. The large detuning between their bare resonance frequencies makes Res #1 and #2 fully independent of each other. In this work, only data obtained from Res #1 are presented.

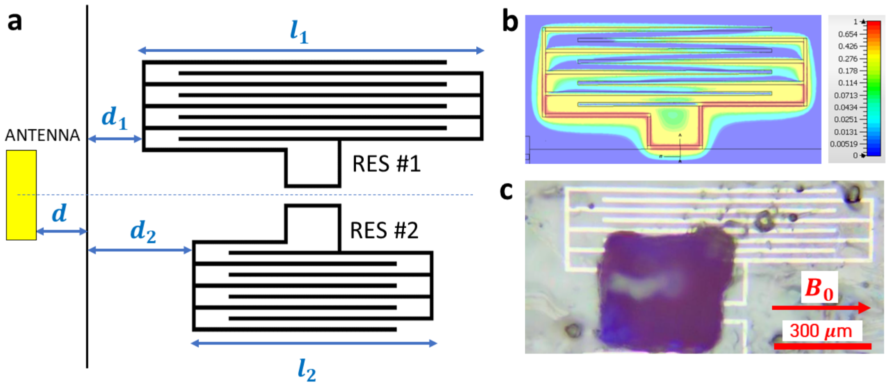

Supplementary Figure 1: **a** Sketch of the two resonators and of their position with respect to the input-output antenna. **b** Electromagnetic simulation of the bare resonator showing the magnitude distribution of the magnetic component of the microwave field. The color scale on the right is normalized to the maximum field value, with red corresponding to maximum and blue to zero. **c** Photo of the planar niobium lumped element resonator with a VOTPP crystalline sample placed on it. The sample has been partially moved to reveal the underlying inductive loop of the resonator. The red arrow indicates the direction of the applied static magnetic field B_0.

The chip is loaded into the same sample holder described in Refs. [3, 4, 5], which is essentially a cylindrical copper waveguide hosting the antenna in its bottom part. The sample holder is cooled inside a Qinu Sionludi dilution refrigerator with a base temperature of 20 mK, and equipped with three-axial superconducting coils and microwave lines and electronics [3, 4]. The chip and the sample holder are oriented so that the main coil generates the static magnetic field in the plane of the resonators (see Fig. 1 of the main text).

2.2 Estimation of the microwave photon number

We estimate the average number of microwave photons, $n_{\rm av}$, in the resonator as reported in Ref. [6], using the following expression:

$$n_{\rm av} = \frac{P_{\rm in} Q_{\rm L,1} 10^{-\frac{\rm IL_1}{20}}}{\pi h \nu_{0,1}^2} \,. \tag{S.12}$$

Here, $P_{\rm in}$ is the input power at the input-output antenna, $Q_{\rm L,1}$ is the loaded quality factor, $\rm IL_1$ is the insertion loss of the resonator Res. #1, $\nu_{0,1}$ its resonant frequency, and h is Planck's constant. The calculation as a function of the input power is shown in Suppl. Fig. 2. The input power values used in this work give an average unitary photon number in the resonator.

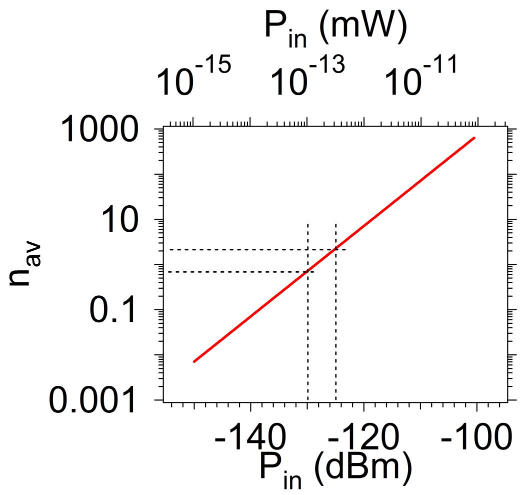

Supplementary Figure 2: Average number of microwave photons in Res. #1 calculated with Eq. (S.12) as a function of the input power at the antenna. Vertical dashed lines show the range of power values used in this work, corresponding to a single photon on average filling the resonator.

Due to the low photon number, the temperature, and the resonant frequency, we estimate the contribution of thermal photons to the photon number. To this end, we consider the resonator as a black body at a given finite temperature, T, and we consider Planck's law:

$$P_{\rm th} = \frac{8\pi h \nu^3}{c^3} \frac{1}{e^{\frac{h\nu}{\kappa_B T}} - 1} \,. \tag{S.13}$$

Equation S.13 gives the power emitted per unit of frequency, ν, unit of solid angle, and unit of surface. Here, h is again Planck's constant, while κ_B is Boltzmann's constant, and c is the speed of light in vacuum. The number of thermal photons per unit

of time, frequency, solid angle, and surface, $n_{\rm th}$, is obtained by dividing Eq. (S.13) by the energy of a single photon, $h\nu$. This leads to

$$n_{\rm th} = \frac{P_{\rm th}}{h\nu} = \frac{8\pi\nu^2}{c^3}\frac{1}{e^{\frac{h\nu}{\kappa_B T}} - 1}\,. \tag{S.14}$$

Figure 3 shows the results obtained with Eq. (S.14) as a function of frequency for different experimental temperature values. The results show that, at the resonant frequency of Res. #1 ($\nu \approx 9.9$ GHz, vertical dashed line), the power associated to the thermal photon emission is much lower than the input microwave one. Moreover, the corresponding number of photons per unit of solid angle and surface is negligible compared to the average number of microwave photons in the microwave tone. Therefore, the effect of thermal photons can be neglected in our measurements.

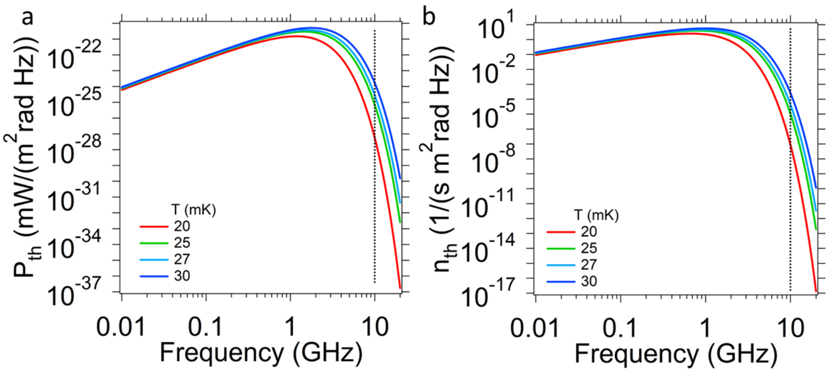

Supplementary Figure 3: **a** Power per unit of frequency, solid angle, and surface as a function of frequency, calculated using Eq. (S.13) for different experimental temperatures employed. **b** Number of photons per unit of solid angle and surface calculated with Eq. (S.14) as a function of frequency. In both **a** and **b**, the vertical dashed line indicates the resonant frequency of Res. #1.

3 Additional Data

3.1 Data for empty resonators

An example of complex reflection scattering parameter (amplitude, $|S_{11}|$, and phase) measured at 30 mK on Resonator Res #1, at microwave power equivalent to single photon level and in zero magnetic field, is shown in Suppl. Fig. 4. The black line is a fit performed using Eq. (3) of the main paper, with $g_\mu = 0$ for any μ.

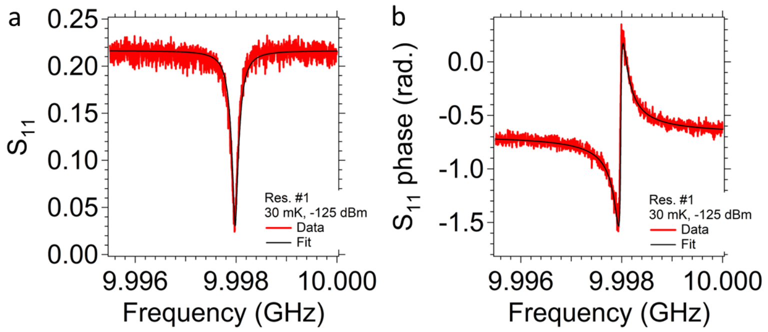

Supplementary Figure 4: Reflection amplitude, **a**, and phase, **b**, measured at 30 mK and single photon powers for a resonator as the one shown in Fig. 1 of main text. Dashed lines are fits based on Eq. (3) of main paper with fixed $g_\mu = 0$ for any μ.

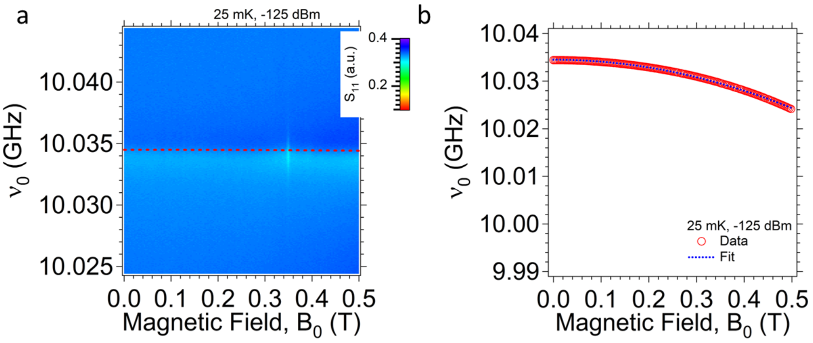

Supplementary Figure 5: 2D reflection map, **a**, and resonant frequency as a function of the static in-plane magnetic field, **b**, for resonator Res. #1. Dashed line is a fit based on Eq. (S.15).

Supplementary Figure 5 shows the reflection map for the empty resonator Res. #1 taken at 25 mK for different applied in-plane magnetic field values. The resonance can be measured at least up to $B_0 = 0.5\,\mathrm{T}$. No signal, except for a very weak one due to magnetic impurities, and no dips with zero reflection are visible in the range in which the resonance with spins is expected ($B_0 \approx 0.34\,\mathrm{T}$). The resonant frequency decreases as a function of the static magnetic field as expected from the magnetic field response of niobium under applied in-plane field. While this change in frequency is almost negligible for the single-ensemble results, it becomes more significant on the multiple ensemble data due to the larger field span used. We take this effect into account by including in our model the dependence of the resonant frequency on the static magnetic field:

$$\omega_0(B_0)/2\pi = \nu_0(B_0) = \bar{\nu}_0 - aB_0^2\,. \tag{S.15}$$

Here, $\bar{\nu}_0$ is the bare resonator frequency at zero field and a a phenomenological parameter. The value of a is obtained by fitting the in-plane dependence of the resonator frequency for an empty resonator (Suppl. Fig. 5).

3.2 Tuning antenna position on BDPA

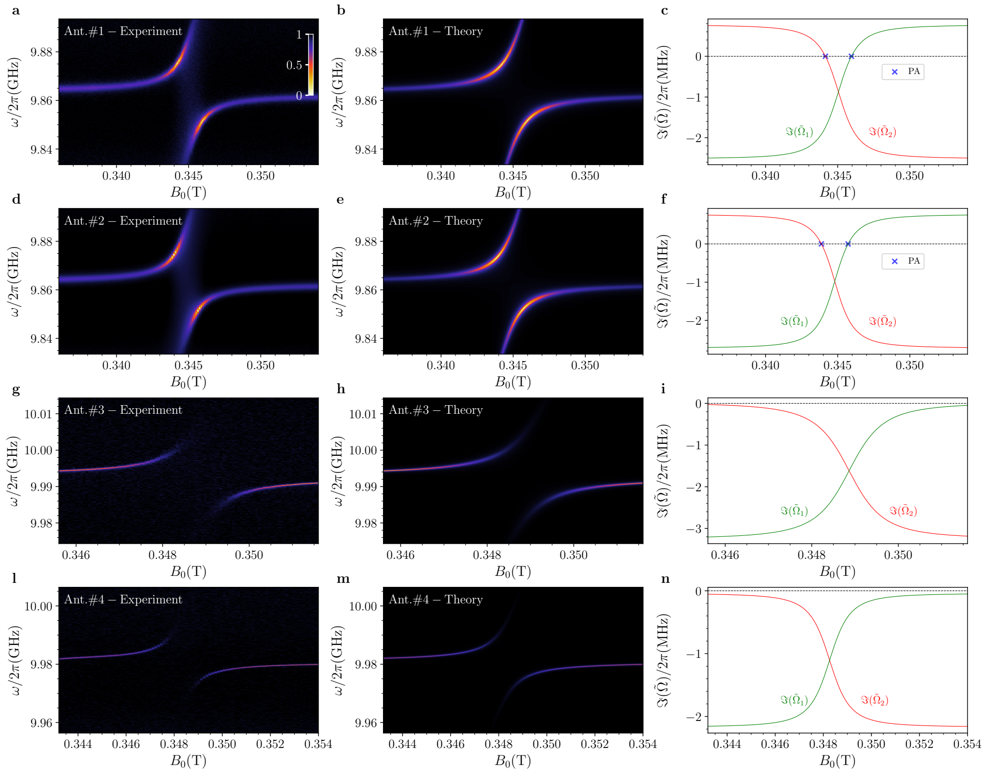

Supplementary Figure 6: **a, d, g, l** Normalized reflection maps ($|S_{11}|$) measured on BDPA for various positions of the input-output antenna, labeled Ant. #1 to Ant. #4. These correspond to decreasing coupling strengths between the antenna and the resonator (i.e., decreasing γ_r). Note that the map for Ant. #1 is identical to the one shown in Fig. 2 (a) of main text. **b, e, h, m** Reflection maps obtained by fitting the data in **a, d, g, l** using Eq. 8 in Methods. **c, f, i, n** Imaginary parts of the eigenfrequencies $\tilde{\Omega}_j$, extracted from the fitted parameters, confirm the presence of perfect absorption in the cases shown in **a, d**, and its absence in **g, i**.

Supplementary Figure 6 shows 2D normalized reflection maps measured for the BDPA sample for different positions of the input-output antenna. The BDPA sample is placed in similar positions over different experiments to obtain strong coupling regimes similar to the one of Fig. 2 of main paper. The position of the antenna is

changed at room temperature before cooldown. We investigate the effect of four different positions, named as Ant. #1 to Ant. #4 in Suppl. Fig. 6, and corresponding to different decreasing coupling strengths between the antenna and the resonator. An avoided crossing is always visible in the 2D map around resonance, as in Fig. 2 of the main paper. The changes in the bare resonator frequency can be attributed to the different cooldown cycles and to the different coupling with the input-output antenna. Specifically, in Suppl. Fig. 6 (a, d), $|S_{11}|$ approaches zero for each polariton, indicating the occurrence of perfect absorption away from the central resonance. This is confirmed by the occurrence of two zeroes in the corresponding imaginary part of the eigenfrequencies, $\tilde{\Omega}_j$ (Suppl. Fig. 6 (c, f)). In contrast, antenna positions Ant. #3 and Ant. #4 (Suppl. Fig. 6 (g, l)) exhibit an anticrossing with higher reflection values and no signature of Perfect Absorption. The corresponding fit results confirm a significant reduction in γ_{r} relative to Suppl. Fig. 6 (a, d) (see tables in Sec. 4). This reduction influences the imaginary parts of the eigenfrequencies $\tilde{\Omega}_j$, giving no zeroes around resonance.

3.3 Additional Data for VOTPP

Supplementary Figure 7 shows the extended data set for the VOTPP crystal. By changing the position of the crystal on the resonator (see Fig. 1 of main text), it is possible to tune the g_μ values and investigate different coupling regimes. Position #A (Suppl. Fig. 7 (a,b,c)) and #D (Suppl. Fig. 7 (l,m,n)) correspond to the results reported in main text, while positions #B and #C show intermediate sample positions. The overall coupling regime is mainly determined by the sample position, while a finer tuning is achieved through the different thermal populations of the lines. Our model perfectly fits all experimental data. Moreover, $\Re(\Omega_j)$ and $\Im(\tilde{\Omega}_j)$ again confirm the correct predictions for the positions and number of nearly zero dips in all experimental datasets, respectively (see the main text for all model details).

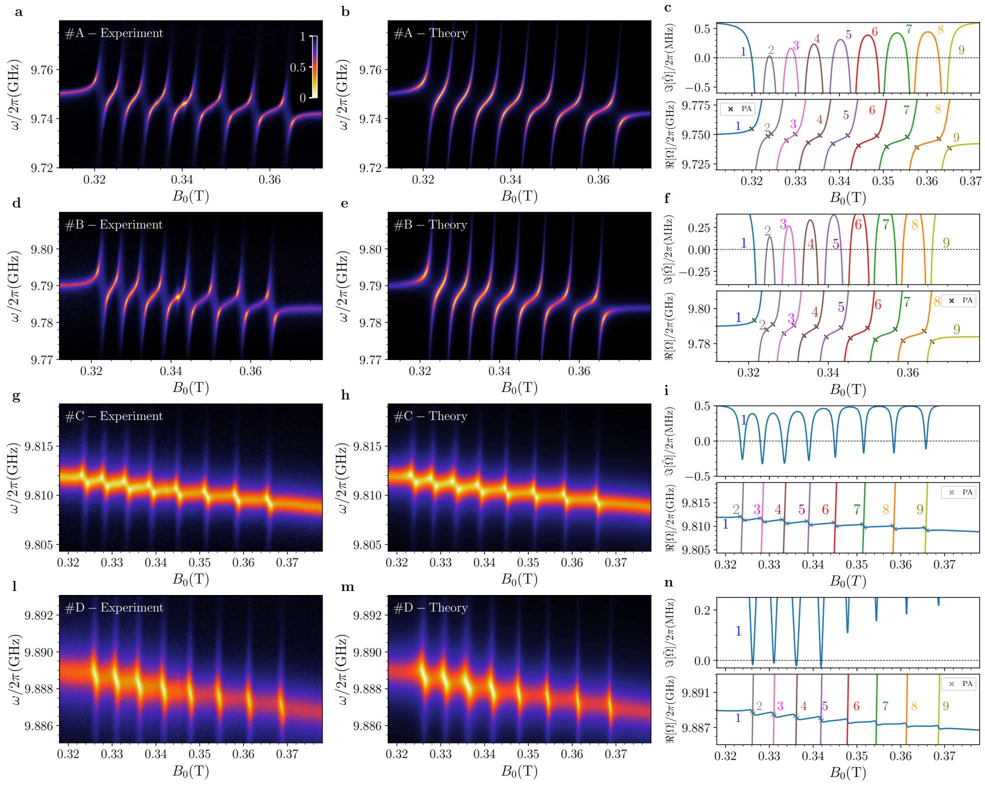

Supplementary Figure 7: Extended dataset showing the results for all four positions of the VOTPP sample on the resonator. Data reported correspond to sample positions #A (**a**,**b**,**c**), #B (**d**,**e**,**f**), #C (**g**,**h**,**i**), #D (**l**,**m**,**n**), respectively (see Fig. 1 of main text). For each position the first columns reports the measured 2D reflection map (**a**,**d**,**g**,**l**), the second column its corresponding fit obtained with Eq. (3) of the main paper (**b**,**e**,**h**,**m**), and the third one shows $\Im(\tilde{\Omega}_j)$ and $\Re(\Omega_j)$ (**c**,**f**,**i**,**n**). All nearly zero experimental dips are correctly predicted at the energies where the imaginary part, $\Im(\tilde{\Omega}_j)$, is zero.

4 Fit Parameters for all data sets

The following tables summarize the fit parameters extracted by means of Eq. (3) of the main paper for all experimental datasets shown in the main text, in Sec. 3.2, and in Sec. 3.3. Each table gives the parameters of the resonator obtained far from resonance and, then, the fit parameters for each μth spin ensemble.

4.1 Parameters for BDPA

Supplementary Table 1 , Suppl. Table 2 , Suppl. Table 3 , and Suppl. Table 4 show the fit parameters for the experimental datasets of BDPA given in Sec. 3.2. The parameters in Suppl. Table 1 are the ones corresponding also to the data shown in Fig. 2 of main text. The coupling strength is larger than the corresponding linewidths, which are associated with the losses of the system, further supporting that BDPA is in the strong coupling regime.

Supplementary Table 1: $S_{11}(\omega)$ fitting parameters for the BDPA dataset shown in Fig. 2 of main text and in Suppl. Fig. 6 (Ant. #1).

Resonator parameters			
a(MHz/T^2)	$\bar{\nu}_0$(GHz)	$\gamma_r/2\pi$(MHz)	$\gamma_{nr}/2\pi$(MHz)
0	9.863	1.67	0.13

Spin ensemble parameters		Coupling strength
g_L	$\gamma_s/2\pi$(MHz)	$g/2\pi$(MHz)
2.042	5.04	20.67

Supplementary Table 2: $S_{11}(\omega)$ fitting parameters for the BDPA dataset shown in Suppl. Fig. 6 (Ant. #2).

Resonator parameters			
a(MHz/T^2)	$\bar{\nu}_0$(GHz)	$\gamma_r/2\pi$(MHz)	$\gamma_{nr}/2\pi$(MHz)
0	9.863	1.64	0.094

Spin ensemble parameters		Coupling strength
g_L	$\gamma_s/2\pi$(MHz)	$g/2\pi$(MHz)
2.044	5.46	19.73

Supplementary Table 3: $S_{11}(\omega)$ fitting parameters for the BDPA dataset shown in Suppl. Fig. 6 (Ant. #3).

**Resonator parameters**			
a(MHz/T^2)	$\bar{\nu}_0$(GHz)	$\gamma_r/2\pi$(MHz)	$\gamma_{nr}/2\pi$(MHz)
0	9.993	0.16	0.056

**Spin ensemble parameters**		**Coupling strength**
g_L	$\gamma_s/2\pi$(MHz)	$g/2\pi$(MHz)
2.047	6.51	12.02

Supplementary Table 4: $S_{11}(\omega)$ fitting parameters for the BDPA dataset shown in Suppl. Fig. 6 (Ant. #4).

**Resonator parameters**			
a(MHz/T^2)	$\bar{\nu}_0$(GHz)	$\gamma_r/2\pi$(MHz)	$\gamma_{nr}/2\pi$(MHz)
0	9.981	0.06	0.132

**Spin ensemble parameters**		**Coupling strength**
g_L	$\gamma_s/2\pi$(MHz)	$g/2\pi$(MHz)
2.048	4.34	12.78

4.2 Parameters for VOTPP

Supplementary Table 5, Suppl. Table 6, Suppl. Table 7, and Suppl. Table 8 show the fit parameters extracted from positions #A, #B, #C and #D, respectively (Fig. 4 of main text and Suppl. Fig. 7). Overall, the coupling strength g_μ of each table decreases from position #A to #D, as a result of the lower amplitude of the resonator microwave field experienced by the spins. Interestingly, for each sample position dataset, the fitted coupling strengths decrease with increasing ensemble (line) index μ, as a result of the different thermal populations of each line, as discussed in the main text. For position #A, all fitted coupling strengths (g_μ) are comparable to their corresponding linewidths, suggesting that the high cooperativity spin-photon coupling regime is achieved. A similar condition is also observed for position #B. Conversely, for position #C and #D each linewidth is significantly larger than its corresponding coupling strength, further suggesting that the weak spin-photon coupling regime is achieved. We attribute this overall increase of the spins decay-rates to the Purcell effect [7, 8]. Specifically, increasing such overlap converts a fraction of the spins decay-rates (spontaneous emission) into an increase of the emission in the resonator mode, which is described by the resonator-spins coupling rates.

Supplementary Table 5: $S_{11}(\omega)$ fitting parameters for VOTPP in position #A, corresponding to the dataset shown in Fig. 4 of main text and in Suppl. Fig. 7 (a,b,c).

**Resonator parameters**			
a(MHz/T^2)	$\bar{\nu}_0$(GHz)	$\gamma_r/2\pi$(MHz)	$\gamma_{nr}/2\pi$(MHz)
66.62	9.754	1.37	0.12

**Spin ensemble parameters**			**Coupling strengths**
μ	$g_{L,\mu}$	$\gamma_{s\mu}/2\pi$(MHz)	$g_\mu/2\pi$(MHz)
1	2.166	13.17	15.92
2	2.135	10.25	15.51
3	2.101	9.28	15.14
4	2.066	10.67	14.70
5	2.029	8.35	14.06
6	1.991	7.74	13.54
7	1.952	8.23	12.99
8	1.913	10.39	12.43

Supplementary Table 6: $S_{11}(\omega)$ fitting parameters for VOTPP in position #B, corresponding to the dataset shown in Suppl. Fig. 7 (d,e,f).

**Resonator parameters**			
a(MHz/T^2)	$\bar{\nu}_0$(GHz)	$\gamma_r/2\pi$(MHz)	$\gamma_{nr}/2\pi$(MHz)
70.93	9.795	1.46	0.15

**Spin ensemble parameters**			**Coupling strengths**
μ	$g_{L,\mu}$	$\gamma_{s\mu}/2\pi$(MHz)	$g_\mu/2\pi$(MHz)
1	2.166	17.40	12.71
2	2.135	13.16	12.33
3	2.102	12.49	12.02
4	2.067	13.12	11.59
5	2.030	12.57	11.12
6	1.993	10.29	10.64
7	1.954	10.60	10.21
8	1.915	12.39	9.81

Supplementary Table 7: $S_{11}(\omega)$ fitting parameters for VOTPP in position #C, corresponding to the dataset shown in Suppl. Fig. 7 (g,h,i).

**Resonator parameters**			
a(MHz/T^2)	$\bar{\nu}_0$(GHz)	$\gamma_r/2\pi$(MHz)	$\gamma_{nr}/2\pi$(MHz)
65.81	9.818	1.59	0.56

**Spin ensemble parameters**			**Coupling strengths**
μ	$g_{L,\mu}$	$\gamma_{s\mu}/2\pi$(MHz)	$g_\mu/2\pi$(MHz)
1	2.165	40.91	3.91
2	2.135	37.97	3.88
3	2.102	43.82	4.17
4	2.068	35.45	3.67
5	2.031	26.26	3.08
6	1.994	24.01	2.83
7	1.955	23.66	2.82
8	1.916	23.80	2.72

Supplementary Table 8: $S_{11}(\omega)$ fitting parameters for VOTPP in position #D, corresponding to the dataset shown in Fig. 4 of main paper and in Suppl. Fig. 7 (l,m,n).

**Resonator parameters**			
a(MHz/T^2)	$\bar{\nu}_0$(GHz)	$\gamma_r/2\pi$(MHz)	$\gamma_{nr}/2\pi$(MHz)
47.76	9.894	1.58	0.45

**Spin ensemble parameters**			**Coupling strengths**
μ	$g_{L,\mu}$	$\gamma_{s\mu}/2\pi$(MHz)	$g_\mu/2\pi$(MHz)
1	2.166	49.18	3.72
2	2.134	45.88	3.52
3	2.102	56.31	3.98
4	2.067	39.12	3.33
5	2.031	28.14	2.50
6	1.993	23.51	2.18
7	1.955	21.23	2.02
8	1.916	21.35	1.94

Supplementary References

[1] Daniel F. Walls and Gerard J. Milburn. Quantum information. In *Quantum Optics*, Ch. 16. (Springer, Berlin, Heidelberg, 2008).

[2] Crispin W. Gardiner and Peter Zoller. *Quantum noise: a handbook of Markovian and non-Markovian quantum stochastic methods with applications to quantum optics.* (Springer Berlin, Heidelberg, 2004).

[3] D. Rieger, S. Günzler, M. Spiecker, A. Nambisan, W. Wernsdorfer, and I.M. Pop. Fano interference in microwave resonator measurements. *Phys. Rev. Appl.*, **20**, 014059 (2023).

[4] D. Rieger, S. Günzler, M. Spiecker, P. Paluch, P. Winkel, L. Hahn, J. K. Hohmann, A. Bacher, W. Wernsdorfer, and I. M. Pop. Granular aluminium nanojunction fluxonium qubit. *Nat. Mater.*, **22**, 194–199 (2023).

[5] K. Borisov, D. Rieger, P. Winkel, F. Henriques, F. Valenti, A. Ionita, M. Wessbecher, M. Spiecker, D. Gusenkova, I. M. Pop, and W. Wernsdorfer. Superconducting granular aluminum resonators resilient to magnetic fields up to 1 Tesla. *Appl. Phys. Lett.*, **117**, 120502 (2020).

[6] Jeremy M. Sage, Vladimir Bolkhovsky, William D. Oliver, Benjamin Turek, and Paul B. Welander. Study of loss in superconducting coplanar waveguide resonators. *J. Appl. Phys.*, **109**, 063915 (2011).

[7] Dengke Zhang, Xiao-Qing Luo, Yi-Pu Wang, Tie-Fu Li, and J. Q. You. Observation of the exceptional point in cavity magnon-polaritons. *Nat. Commun.*, **8**, 1368 (2017).

[8] Xufeng Zhang, Chang-Ling Zou, Liang Jiang, and Hong X. Tang. Strongly coupled magnons and cavity microwave photons. *Phys. Rev. Lett.*, **113**, 156401 (2014).
